# Phytonanotechnology applications in modern agriculture

**DOI:** 10.1186/s12951-021-01176-w

**Published:** 2021-12-20

**Authors:** Meng Jiang, Yue Song, Mukesh Kumar Kanwar, Golam Jalal Ahammed, Shujun Shao, Jie Zhou

**Affiliations:** 1 College of Agriculture and Biotechnology, Zhejiang University, Yuhangtang Road 866, Hangzhou, 310058 People’s Republic of China; 2 Institute of Crop Sciences, National Key Laboratory of Rice Biology, Zhejiang University, Yuhangtang Road 866, Hangzhou, 310058 People’s Republic of China; 3 Department of Horticulture, Zhejiang Provincial Key Laboratory of Horticultural Plant Integrative Biology, Zhejiang University, Yuhangtang Road 866, Hangzhou, 310058 People’s Republic of China; 4 Key Laboratory of Horticultural Plants Growth, Development and Quality Improvement, Agricultural Ministry of China, Yuhangtang Road 866, Hangzhou, 310058 People’s Republic of China; 5 College of Horticulture and Plant Protection, Henan University of Science and Technology, Luoyang, 471023 People’s Republic of China

**Keywords:** Agricultural systems, Agrochemicals, Crop breeding, Growth and development, Nanotechnology, Postharvest preservation

## Abstract

With the rapidly changing global climate, the agricultural systems are confronted with more unpredictable and harsh environmental conditions than before which lead to compromised food production. Thus, to ensure safer and sustainable crop production, the use of advanced nanotechnological approaches in plants (phytonanotechnology) is of great significance. In this review, we summarize recent advances in phytonanotechnology in agricultural systems that can assist to meet ever-growing demands of food sustainability. The application of phytonanotechnology can change traditional agricultural systems, allowing the target-specific delivery of biomolecules (such as nucleotides and proteins) and cater the organized release of agrochemicals (such as pesticides and fertilizers). An amended comprehension of the communications between crops and nanoparticles (NPs) can improve the production of crops by enhancing tolerance towards environmental stresses and optimizing the utilization of nutrients. Besides, approaches like nanoliposomes, nanoemulsions, edible coatings, and other kinds of NPs offer numerous selections in the postharvest preservation of crops for minimizing food spoilage and thus establishing phtonanotechnology as a sustainable tool to architect modern agricultural practices.

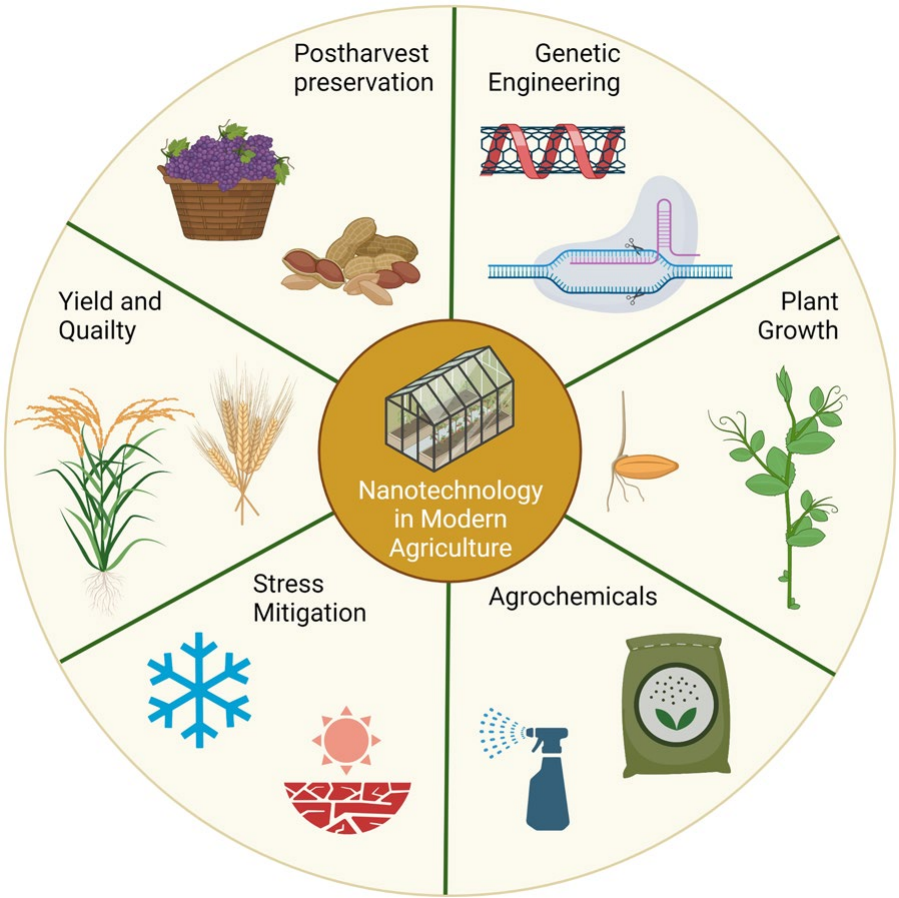

## Introduction

Globally, the agricultural systems are being challenged with more and more unpredictable hazards. To safeguard sustainable agriculture and food production, the advanced agronomic application of nanotechnology in plants, termed phytonanotechnology, is of great significance [1]. Phytonanotechnology can improve agricultural production by minimizing relevant losses and increasing the efficiency of inputs, thus providing an important solution for keeping the feasible development of agro-systems and related sectors [2]. The prospective use of phytonanotechnology can bring a revolution in the agro-systems, through an enhancement in crop yields and productivity, while maintaining environmental sustainability, and ecological and economic stability [3]. The applications of phytonanotechnology in agricultural systems have helped the progress of ‘intelligent’ cropping and promoted conventional agricultural ways and practices, offering more environmentally efficient, and ingenious management [4]. Compared with the production materials used in traditional agricultural practices, phytonanotechnology offers many uses and scopes to be developed and understood.

Nanomaterials (NMs), have internal surface structures or external dimensions with three or two dimensions ranging from 1 to 100 nm [5]. NMs have special physicochemical characters, such as enhanced reactivity, atypical surface structure, and high surface-to-volume ratio which differ individually from those of their molecular counterparts [6]. NMs also offer multifunctional, programmed, self-regulated, target-specific, and time-controlled abilities [7, 8]. Owing to these special and versatile physicochemical characters, NMs are utilized gradually in a large number of agricultural practices. In detail, NMs participate in the targeted-specific transfer of proteins, nucleotides, or other phytoactive molecules that can genetically regulate and modify the metabolism in crops. As special carriers of agrochemicals, NMs can provide a larger specific surface area to herbicides, fertilizers, and pesticides and ensure their ‘on-demand’ release, whether it is for preventing pathogens, pests, and diseases, or nutritional needs [9]. Thus, NMs can promote controlled and targeted nutrient delivery, resulting in enhanced crop growth and development.

Numerous nanoparticles (NPs) have been widely utilized in phytonanotechnology, such as mesoporous silica NPs (MSNs), carbon nanotubes (CNTs), quantum dots (QDs), magnetic NPs (MNPs), metallic NPs, and metal oxide NPs [1]. MSNs include honeycomb-like porous structures with tunable outer particle diameter and tunable pore size in the nanometer range. They have hundreds of empty channels that are capable of absorbing or encapsulating different bioactive molecules or agrochemicals. Plasmids containing the Green Fluorescent Protein (GFP) gene can be delivered by MSNs, together entering into plant cells and finally triggering the expression of the target gene [10]. The enzyme or protein loaded by the system of MSNs can be used for genome modifications or biochemical analysis in plants [11]. This procedure avoids the delivery of the reformed characters to the next generations by integrating the transgene into the genome. CNTs are the allotropes of carbon that have cylindrical nanostructures with diameters between 1 and 50 nm [12]. They are classified as multi-walled nanotubes (MWNTs) and single-walled nanotubes (SWNTs). QDs are nanocrystals of semiconductor materials with diameters between 2 and 10 nm [13]. They can generate distinctive fluorescence that can be utilized for subcellular imaging or labeling. MNPs comprise different magnetic materials, e.g., Cobalt (Co), Nickel (Ni), Iron (Fe), and their derivative compounds. They are categorized as magnetic virus-like NPs (VNPs) [14], carbon-coated MNPs [15], and other magnetic NPs. They can be operated by using magnetic field gradients for targeted delivery. Au and Ag NPs are the most commonly used metallic NPs due to their better effectiveness in delivering biomolecules in crops [3]. Likewise, metallic oxide NPs of ZnO, CuO, SiO_2_, and TiO_2_ NPs, have also been broadly utilized as a delivery carrier in plants system due to the greater light absorption, catalytic, and electrical characteristics [16]. Numerous metallic and metallic oxides NPs have been applied in diverse crop management procedures including fertilization and crop protection [17]. Despite all the beneficial effects, attention must be paid to design safety principles to address the community dealing with the possible opposing influences of new NMs on the ecosystem (for example, application of NMs in a daily necessity product) [18].

**Fig. 1:**
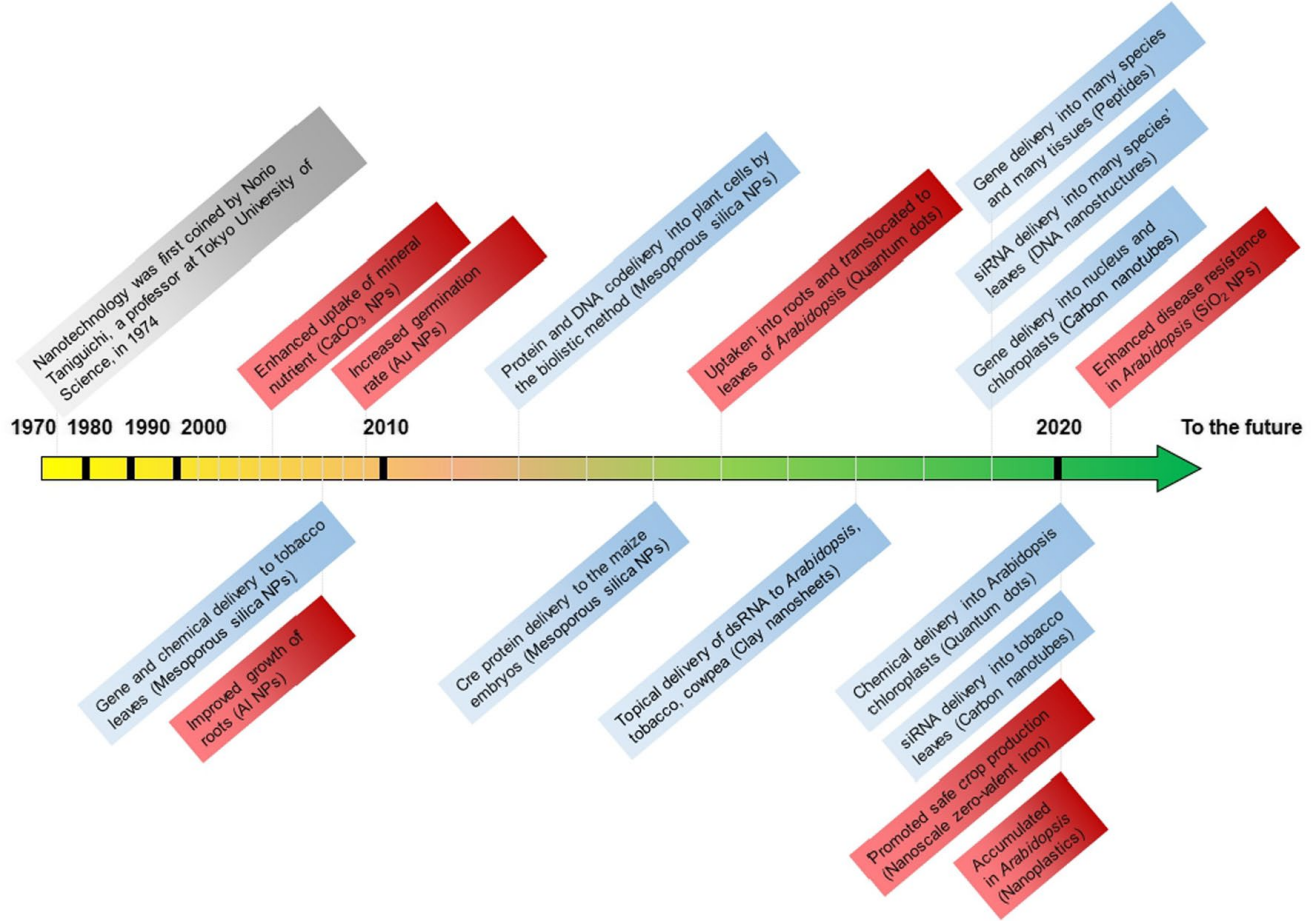
Timeline of nanotechnology applications in agriculture. The applications of nanotechnology in genetic engineering and crop breeding were shown in blue boxes, and the applications of nanotechnology in crop growth were shown in red boxes

**Fig. 2:**
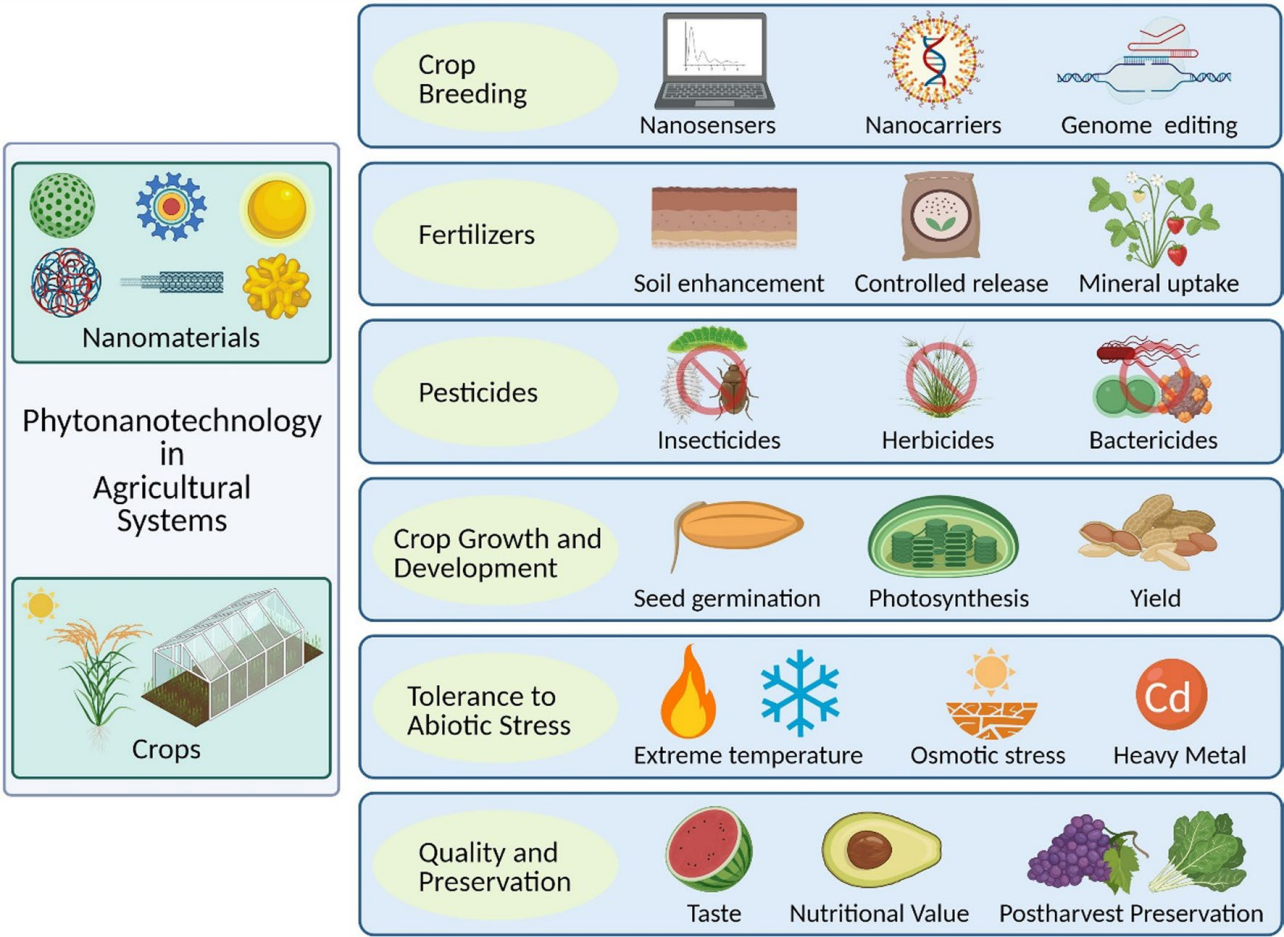
Schematic illustrations of the applications of nanotechnology in agricultural systems

Since the related research of phytonanotechnology in agricultural systems is exponentially increasing (Fig. 1), a complete review outlining the novel features of phytonanotechnology and its roles in crop development and other agricultural production is imperative. Thus, this review is aimed to provide the readers with complete mechanistic insights into the new paradigms of phytonanotechnology progressions in agriculture by investigating their roles in crop breeding, agrochemicals delivery, crop growth and development, and other allied functions to sustain and design a better agriculture system for the future (Fig. 2).

## Special properties and surface modifications of NPs

The special properties of NPs make them more effective than ordinary materials, e.g., ions and molecules [19]. In a greenhouse experiment, crops were collected after growing for 80 days in soil modified with CuSO_4_ or CuO particles (bulk or nano) [20]. The Cu-content in the root of nano-CuO treated crops was higher compared with bulk CuO, CuSO_4_, or control. This indicated that nano-CuO was more easily absorbed by crops. Time-resolved tests were performed to analyze the physiological effects and the translocation of Zn in the common bean (*Phaseolus vulgaris* L.) [21]. The absorption spectroscopy of near-edge X-ray exhibited that ZnSO_4_ and ZnO (300 nm) were more difficult to be absorbed by roots of bean than ZnO (40 nm). It further demonstrated that ZnO in the nano state with smaller size was more easily absorbed by the crops. The highest level of Zn was found in ZnO NPs sprayed seedlings with ~ 78-fold in comparison to ~ 27-fold detected in ZnSO_4_ treated plants [22]. It is inferred that the higher Zn accumulation with ZnO NPs treatment was reasoned by the size property or better adhesion of the nano form compared to the ionic form, causing their more effective entry into the seedlings [23, 24]. The different transportation and accumulation features of silver (Ag) NPs and Ag^+^ were reported in rice (*Oryza sativa* L.) [25]. Ag NPs are translocated and assimilated more validly on the root surface compared to Ag^+^. Briefly, NPs are lightly transported in crop seedlings, so the optimum dosage of suitable size of NPs enhances their absorption in crops [26]. The activity, aggregation, catalytic charge, crystallinity, porosity, or redox potential can influence the uptake of NPs in crop seedlings. Furthermore, the high surface activity of NPs may speed up the metal component release [27]. The electrostatic attraction, hydrophilicity, lipophilicity, and physical adsorption of NPs, influence the accumulation [28]. For instance, the NPs with neutral or positive charges are more beneficial for agglomeration, but the NPs with negative charges are more beneficial for translocating in crops [29].

The absorption of NPs by crops can be enhanced by modifying their surface using numerous objects, such as iron, aminopropyl triethoxysilane, fluorescein isothiocyanate, natural organic matter [29], humic acid [30], polyvinylpyrrolidone [31], citrate [26], or polyethylene glycol [32]. In detail, the hydrophilic protective sheet makes NPs easily penetrable to crops [33], while the protein-encapsulated NPs are more stable in crops [34]. Nanocarriers allow the assimilation and carriage of nutrients such that nano-liposomes aid the transport and absorption of nutrients in various crops [35]. Organic macromolecules such as chitosan decrease the agglomeration of NPs, increase the stability of NPs, and allow them effortlessly enter into epidermal cells of leaves by modifying their chemical and physical characteristics [36]. Moreover, the NPs surface charge can be improved by coating, which contributes to their translocation [37]. Recent reports found that the material of surface coating can prevent the closure of stomata by decreasing the indiscriminate gathering of NPs, thus enhancing the absorption of NPs in crops [38]. Moreover, the utilization of functional groups and surfactants improves the bioavailability of NPs by increasing the NPs adhesion on the surface of leaves [39, 40]. For instance, hydroxyapatite can be used to alter the surface of NPs to induce the absorption of leaves and decrease the aggregation of NPs [30].

## Nanotechnology in genetic engineering and crop breeding

**Fig. 3:**
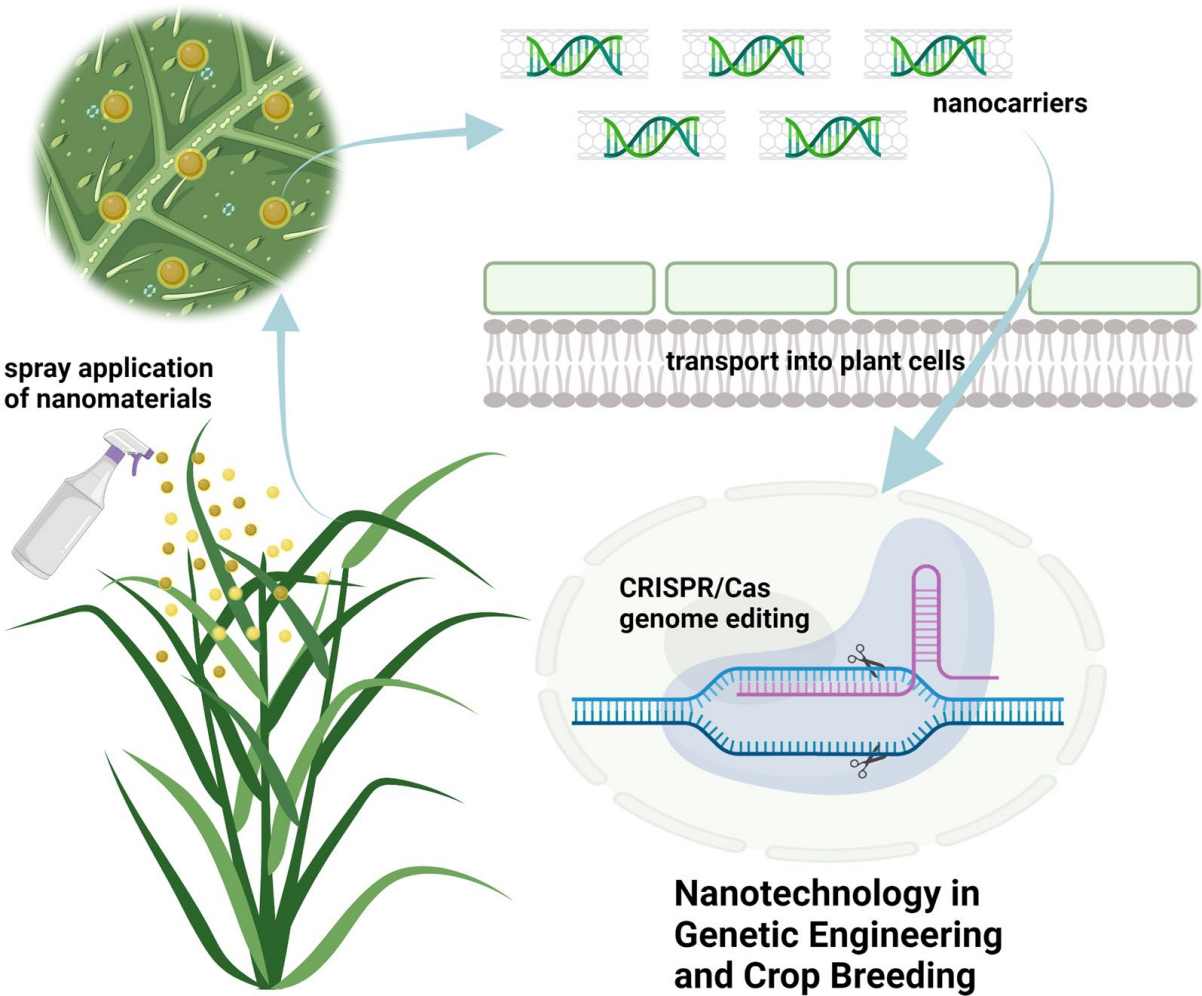
Schematic diagram of the applications of nanotechnology in genetic engineering and crop breeding. Plasmids containing genes that encode Cas and the sgRNA are delivered into the plant cell through *Agrobacterium*-mediated transformation, protoplast transfection, particle bombardment, or even spray application with NMs. The CRISPR/Cas genome-editing system consists of the Cas endonuclease, which can site-specifically cleave double-stranded DNA, and an sgRNA that hybridizes to about 20 nucleotides of the target sequence

Crop breeding is acknowledged as a technology to improve the genetic characteristics of crops by generating high-yield and high-quality varieties [41]. Several conventional and molecular approaches have been used in crop breeding, including functional genomic tools, genetic selection, mutagenic breeding, physical maps, somaclonal variations, and whole-genome sequence-based approaches [42]. Nanotechnology is a new pioneering approach to improve the efficiency and accuracy of crop breeding (Fig. 3).

Nanobiotechnology improves the efficacy of crop breeding by delivering exogenous biochemicals or nucleotides into plant cells [43]. As transportation across the cell wall and limited size of inserted genetic material signify main barriers to transport of external molecules into crop cells, the combination of biotechnology and nanotechnology offers more chances as novel means of biomolecule transport into the cells through the cell wall [44]. Recently, a nanomaterial-mediated gene delivery system has been developed. High transformation efficiency is achieved without external physical or chemical means in plant cells, showing excellent applications in plant genetic engineering [45]. For example, the foremost application of delivering NMs into crops was carried by Torney et al. [10], where MSNs transported a target gene into the leaves of tobacco (*Nicotiana benthamiana* L.). SiO_2_ NPs have been developed to transport DNA to crops, e.g., tobacco and maize (*Zea mays* L.) with no adverse effects [46]. The technology of particle bombardment (or gene-gun) has been used to transform plants since the early 1980s, using either tungsten or gold particles as DNA carriers [47]. The current system is adapted to allow NPs delivery, which most likely decreases the cell damage caused by microprojectile hits during the bombardment and thus improves the expression efficiency of the transgenes. The DNA-coated NPs are utilized as bullets in the gene-gun technology to bombard the tissues or cells to deliver the desired genes into the target crops [48]. Silicon carbide-participated plant transformation has been found to transfer the sequences or fragments of DNA in various *calli* [maize, tobacco, rice, soybean (*Glycine max* L.), and cotton (*Gossypium hirsutum* L.)] as an effective method [49]. The complex of MNPs and β-glucuronidase target gene was permeated to the pollens of cotton by magnetic force, with no negative effects on the viability of pollens. By pollinating with magnetofected pollens, the transgenic cotton plants were effectively selected and exogenous genetic information was steadily inherited into offspring achieved by selfing, successfully combined into the genome, and finally expressed [50]. The scaffolds of carbon nanotubes were applied to deliver linear or plasmid DNA, in cotton, tobacco, and wheat (*Triticum aestivum* L.) leaves, causing a strong transient expression of GFP [51, 52]. In addition to the above-mentioned DNA delivery, NPs are also used to deliver RNA into plant cells. Chitosan NPs-embedded small interfering RNA (siRNA) delivery systems have offered a novel strategy for crop improvement by permitting the unique dominance of the target pest as chitosan has the capability to validly bind with RNA and the ability to penetrate cell membranes [53]. The double-stranded RNAs (dsRNAs) carried on non-toxic and degradable clay nanosheets offer defense against cauliflower mosaic virus in leaves of tobacco [54]. The siRNA was transferred to tobacco seedlings constitutively expressing the GFP gene, resulting in a high percentage silencing of the target gene [51, 52]. The carbon nanotubes-mediated platform realized effective RNA transfer into intact crop cells and protected RNA from nuclease degradation, enabling gene silencing of endogenous GFP in mutants [52].

Gene editing has been broadly used in crop science and has a great possibility of becoming the ‘game changer’ in crop breeding [55]. The system of clustered regularly interspaced short palindromic repeats (CRISPR)/CRISPR-associated proteins (Cas), an RNA-based guard organization in prokaryotes, containing the Cas proteins and CRISPR repeat spacer arrays, has effectively been utilized for genome editing in crops [56]. CRISPR/Cas genome editing has been useful in plants by traditional transformation and regeneration processes [55]. Delivery, low HDR efficiency, species dependence, and tissue culture and regeneration are the four main challenges in genome editing of crops. With the characteristics of small size, differently charged, high-throughput, and high tensile strength, NMs can enhance the specificity and efficiency of the CRISPR/Cas9 technique and minimize the possibility of off-target [57]. Contemporary developments in NMs-mediated particular transport of CRISPR/Cas9 single guide RNA (sgRNA) have started a novel period of genetic engineering. Moreover, MSNs have been applied to transport Cre recombinase in the immature embryos of maize as carriers, loading the sites of *loxP* site recombined into the chromosomal DNA. The *loxP* fragment was acceptably integrated after the delivery of modified MSNs in crop cells [11]. Cationic arginine gold NPs gathered Cas9En (E-tag)-ribonucleoproteins (RNP) transport of sgRNA in cultured cell lines offered high efficiency (around 30%) of active nuclear or cytoplasmic gene editing, which can significantly promote the study of crops [58]. Although current nanotechnology has promoted the CRISPR/Cas9 technique in numerous crops, phytonanotechnology-based approaches are also demanded to overcome other difficulties to the genome editing of crops. There are still a lot of challenges, e.g., the range of plant species that can be genetically engineered, the forms of CRISPR/Cas genome editings that can be powerfully utilized in crops, the labor and time efforts essential for crop regeneration, and the low transport efficiency.

## Phytonanotechnology applications during the lifecycle of crops

NMs, such as MSNs, Au NPs, SiO_2_ NPs, and Chitosan NPs are informed to enhance crop growth and development from the initial phases of seed germination to death or senescence in numerous crop species including soybean, rice, wheat, peanut (*Arachis hypogaea* L.), tomato (*Solanum lycopersicum* L.), potato (*Solanum tuberosum* L.), and onion (*Allium cepa* L.) [59]. NMs exhibit positive effects on crops by accelerating crop breeding, promoting seed germination, increasing photosynthesis, enhancing mineral uptake, and improving crop quality and yield [60]. The application of nanotechnology to the overall growth and development process in plants is primarily dependent on the concentration, composition, size, and chemical and physical characteristics of NMs [61].

### Seed germination

**Fig. 4:**
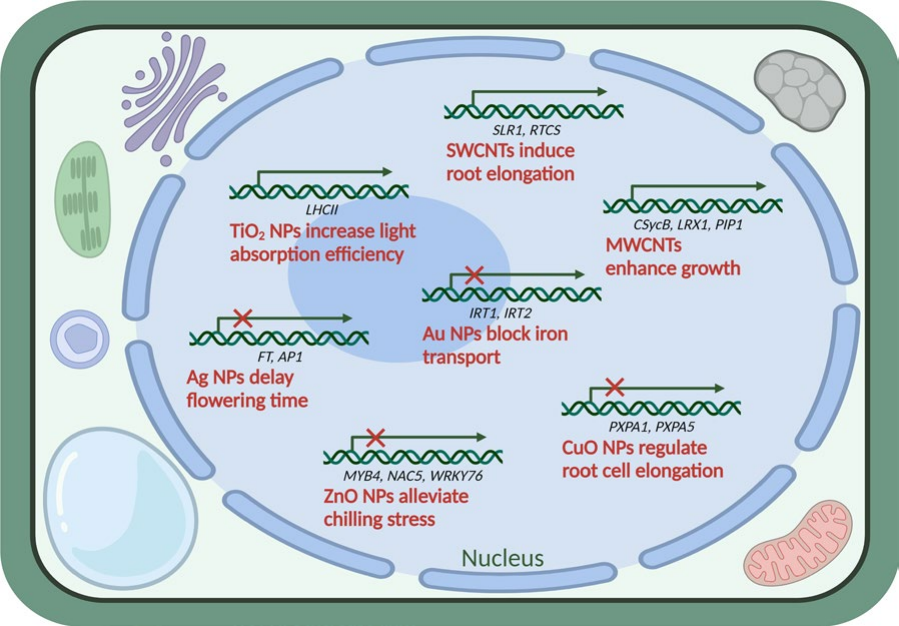
Molecular mechanisms of nanomaterial actions in plant cells

Seed germination is the first step and the most sensitive period in the life cycle of plants [62]. Numerous studies have revealed that the application of nanotechnology has beneficial influences on the germination of seeds. Studies have shown that NMs increase water absorption and utilization and have the ability to penetrate the seed coat, which can eventually improve seed germination and seedling growth by stimulating the enzyme system [63–65]. For instance, the application of Zn NPs significantly promotes the germination of seeds in different crop species, e.g., onion, soybean, peanut, and wheat [66, 67]. The use of metal oxide NPs such as TiO_2_ and SiO_2_ NPs for seed treatment, was found to substantially improve seed germination in several crops [62]. Additionally, multiwalled carbon nanotubes (MWCNTs) applications also facilitate the germination of seeds in crops, such as soybean, maize, peanut, wheat, tomato, garlic (*Allium sativum* L.), and barley (*Hordeum vulgare* L.) [68–70]. At the molecular level (Fig. 4), single-walled nanotubes (SWCNTs) upregulated the expression of *SLR1* and *RTCS* genes in maize root tissues and increased root growth [71]. Although a large number of studies on the positive interactions between NMs and the germination of the seeds are being reported in crops, the underlying mechanisms of the superiority of NMs to traditional materials in seed germination remain hitherto unknown and need more comprehensive assessment.

### Photosynthesis

Photosynthesis is a vital process for the growth and development of plants. It transforms light energy into chemical energy [72]. Thus, enhancing the efficiency of photosynthesis is critical for the better growth of crops [73]. It has been reported that NMs can enhance the system and enzyme activity of photosynthesis and the content of chlorophylls, which can eventually improve the overall plant growth [74]. At the physiological level, the nano-anatase TiO_2_ application enhances the rate of photosynthesis by stimulating the Rubisco enzyme activity, which could ultimately enhance the growth and development of crops [75]. The SiO_2_ NPs increase the capacity of photosynthesis via enhancing the photosynthetic metabolism and the carbonic anhydrase enzyme activity in crops [76]. The highest photosynthetic ability and an increase in chlorophyll content were found, when the seedlings were treated with 0.5 g/L of SiO_2_ NPs in wheat [77]. A comparable effect was also found by Rafique et al. [78], which showed that 60 mg/kg of TiO_2_ NPs (< 20 nm) increased the content of chlorophylls by 32.3% compared to the control in wheat. At the molecular level (Fig. 4), TiO_2_ NPs induce the *light-harvesting complex II* (*LHCII*) gene in *Arabidopsis thaliana*, and finally enhance the chloroplast light absorption efficiency and thylakoid membrane LHCII content [79]. It has been reported that the Si NPs can stimulate the *PsbY* and *HemD* gene expression related to the biosynthesis of chlorophyll, thereby resulting in a higher photosystem II activity and an increased chlorophyll content [80, 81].

### Yield

NPs can influence the yield of crops by changing the physiology and biochemistry of plants. In detail, Fe_3_O_4_ NPs maintain iron homeostasis, decrease lipid peroxidation, and induce ferritin content in maize [82]. Crops grown in the soil modified (hydrophilic or hydrophobic coating) or unmodified with nano-TiO_2_ for 2 months displayed that inorganic carbohydrate production, mineral uptake (P, Se, Mn, Fe, Mg, Ca, and Cu), chlorophyll, enzyme activity, and plant growth were enhanced significantly with the application of coated NPs [83]. Similarly, foliar applications of Mn, Fe_2_O_3_, and Mo NPs also increase crop yield [28]. Moreover, various NPs have been used to enhance the dietary value and nutritional components of fruits or food grains. Foliar application of Cu NPs increases antioxidant enzyme activity, fruit firmness, and vitamin content, thus enhancing fruit freshness and quality in tomatoes [84]. At the molecular level (Fig. 4), Ag NPs affect the expression of *VIN3*, *VRN2*, *FRI*, and *FLC* in the vernalization pathway, and cause the down-regulation of key flowering control genes *AP1* and *FT*, thereby delaying flowering [85]. The application of different NPs influences the quality and yield of crop seeds or fruits, which completely depends on the mode of treatment, property, size, and type of NPs used. Further investigation on dose-dependence, long-term exposure effects, as well as molecular studies such as metabolomics or proteomics can be an access device in determining the precise function of NPs on the quality and yield of seeds or fruits.

### Quality

The significance of phytonanotechnology in crop-derived foods can be judged by evaluating their functions in the development of crop products in the fields of food quality, e.g., taste and nutritional value. The application of nanotechnology offers numerous ways to improve food taste. As compared to the larger materials which usually degrade substances over a longer time, the smaller NPs deliver favorable ways of enhancing the food bioavailability because the nearly subcellular size contributes to a higher level of bioavailability. Several metallic oxide NPs, e.g., SiO_2_ and TiO_2_, have been utilized as flow or color stuffs in food products [86]. Dekkers et al. [87] reported that SiO_2_ NPs were used as applied-NM to deliver flavors or fragrances into the food items. The novel technology of nano-encapsulation has been utilized widely to provide the balance of culinary and to increase flavor preservation and release [88]. The encapsulation of ferritin nanocages can increase the thermal stability and solubility of ferritin obtained rutin [89].

Large numbers of bioactive substances e.g., vitamins, proteins, carbohydrates, and lipids are more sensitive to the enzyme activity of the duodenum and stomach, and the high acidic environment. Nano-encapsulation permits the bioactive substances to assimilate readily in food products and allows them to struggle with such opposing conditions, which is difficult to attain in the non-capsulated forms because of the low water-solubility of the bioactive substances. The nano-emulsification, nano-structuration, and nanocomposite are the diverse tools that have been used to encapsulate the compounds in tiny shapes to more efficiently transport nutrients like antioxidants or proteins for health or nutritional assistance. Polymeric NPs are reported to be appropriate for bioactive substances encapsulation (such as vitamins or flavonoids) to transport and protect the bioactive substances for different purposes [90]. NPs-based tiny edible capsules are being made to develop the delivery of fragile micronutrients, vitamins, and medicines of the daily use food item and thus, offer an important advantage for human health [91].

### Postharvest preservation

Food safety has become a worldwide issue because of increasing food demand and compromised crop yields resulting from climate change, soil degradation, and crop disease proliferation [92]. As per estimates, the global population will reach 9.6 billion by 2050, consequently, the demand for staple crops will increase by 60% [93]. Nanotechnology, such as nanofabrication and nanoencapsulation, can provide new added value solutions for the fortification of foods with bioactive and targeted controlled release in the gut to compensate for the food demand–supply objective [94].

A required packaging material must have moisture and gas permeability joint with biodegradability and strength [95]. As microcapsulization of vegetable oils have been used in postharvest preservation for a long time, nano-faciliated “active” and “smart” food packagings provide numerous advantages over conventional techniques with improved antimicrobial films, barrier properties, and mechanical strength [96]. Nanomaterials can also support food preservation aspects by being added directly into a food matrix or food contact materials such as packaging. The increasing application of nanotechnology offers numerous selections to extend the shelf-life of food for longer use. The use of nanotechnology in food preservation has revealed beneficial effects on diminishing spoilage. Nanoliposomes, nanoemulsions, edible coatings, and other different kinds of NPs have been widely used in the postharvest preservation of crop-derived foods [97].

Nanoliposomes have been certified to be valid in the targeted transport of antimicrobial compounds, nutraceuticals, nutrients, and vitamins owing to the small size with a larger area for surface contact [98]. It has been found that many factors are influencing the fusion of liposomes with bacterial cells, like bacterium surface pH, temperature, divalent cations, and characteristics of the bacterial membrane. The delivery of negatively charged liposomes into the cells is promoted by nonspecific receptors, followed by particle recognition, adhesion and ultimately causing endocytosis [99]. The lipid composition of the bacterial membrane plays an important role in the fusion of liposomes to the bacterium [100].

Nanoemulsions are the mixtures of different immiscible liquids that are stabilized by a surfactant (e.g., modified starches, proteins, and lipids) with the average droplet size (20 to 200 nm). The food-grade surfactants like lecithins, sugar esters, and polysorbates emulsifying agents utilized in postharvest preservation of crops play key roles in the stabilization of nanoemulsions through adequate responses to environmental stresses, such as enhanced loading capacity, steric hindrance, and the repulsive electrostatic interactions [101]. The characteristics of nanoemulsions cause them appropriate intermedia for transport of hydrophobic drugs, bioactive molecules, and nutraceuticals to encapsulate hydrophobic antioxidant ingredients [102].

Edible coatings have been reported to extend the shelf-life of perishable crop-derived foods, particularly vegetables or fruits. Edible coatings are traditionally thin materials applied with food-grade substances that are utilized on the surface of vegetables and fruits, therefore becoming a portion of crop-derived foods that stay on the outer surface during consumption and use [103]. The edible coatings contribute to postharvest preservation by controlling carbon dioxide and oxygen permeability, decreasing moisture loss, thus decreasing the rates of solute respiration, oxidation, and migration without compromising the quantity or quality of the crop-derived foods [104]. Edible coatings have good performance to play as a carrier vehicle for antimicrobial agents, micronutrients, flavoring compounds, colored pigments, and anti-browning mediums that assist in increasing valid period of crop-derived foods by preventing the survival of the pathogens on the outer surface of fresh vegetables and fruits during the food spoilage [105].

Nanocomposites have been widely applied for food packaging and preservation because of their antifungal or antibacterial properties [97]. The antimicrobial characteristic owned by Ag NPs has been widely used for postharvest preservation owing to their inactivation of food spoilage microorganisms. Special properties of Ag such as low volatility and stability at high temperatures and toxicity against various types of microbes make it a beneficial choice for application in postharvest preservation [106]. Nanocomposites applied with Ag NPs have been utilized to prolong the shelf-life of different kinds of crop-derived foods [107]. Mechanism of Ag NPs action is mediated by the release of Ag^+^ in the following steps: adhesion to the cell surface of pathogens, disruption of the cell membrane of pathogens, damage of DNA, and cell death [108]. The primary mechanism of Ag NPs has been extracted in three routes: interruption of ATP production and DNA replication by the Ag^+^ uptake, production of reactive oxygen species (ROS) by Ag NPs and Ag^+^ in the cells, and finally deterioration of the membrane of cells by Ag NPs [108]. Because of the excellent antimicrobial characteristic against microorganisms, TiO_2_ NPs are extensively utilized in postharvest preservation [109]. Photocatalysis of TiO_2_ inactivates food spoilage bacteria by inducing the lipid peroxidation of phospholipids in microorganisms’ cell membranes [110]. The antimicrobial mechanism of TiO_2_ NPs can be described in three routes; ROS production upon the activation by the UV and visible wavelength of light, oxidative stress and lipid peroxidation in cells, and the death of cells [111]. Despite these, more studies are needed to explore the underlying mechanism of the interactions between NPs and crops to increase the shelf-life and delay the ripening.

## Potential applications of phytonanotechnology in stress mitigation

The change in climate around the world results in extreme temperature, salinity, drought, and various environmental pollution with excessive heavy metals are thought as one of the main reasons that influence the growth and development of crops [112, 113] (Table 1). The augmented adaptation of crops needs a many-sided approach, e.g., regulation of hormones, activation of plant enzymatic system, expression of stress genes, avoidance of water deficit stress, and control of the heavy metal translocation and uptake [114, 115]. Advances in NMs can raise the production of crops in the present opposing environment [59, 116]. The studies below display that nanotechnology can alleviate the bad influences of abiotic stress.

**Table 1:** Ameliorative effects of NMs on abiotic stress in crops

Abiotic stress	Nanomaterials (size)	Plant species	Ameliorative effects	References
Heat	MWCNTs (10–35 nm)	Tomato (*Solanum lycopersicum* L.)	Upregulated the expression of various stress-related genes including HSP90	Khodakovskaya et al. [117]
	CeO_2_ NPs (~ 10 nm)	Maize (*Zea mays* L.)	Decreased production of hydrogen peroxide (H_2_O_2_) and upregulation of *HSP70*	Zhao et al. [118]
	TiO_2_ NPs (~ 16 nm)	Tomato (*S. lycopersicum* L.)	Enhanced photosynthesis, regulated energy dissipation, and induced stomatal opening	Qi et al. [119]
	Ag NPs (10–20 nm)	Wheat (*Triticum aestivum* L.)	Protected plants against heat stress and improved plant growth significantly	Iqbal et al. [120]
	Ag NPs (15–30 nm)	Wheat (*T. aestivum* L.)	Alleviated the harmful effects of salinity stress	Abou-Zeid and Ismail [121]
	Se NPs (10–40 nm)	Tomato (*S. lycopersicum* L.)	Increased chlorophyll content, hydration of plants, and growth	Djanaguiraman et al. [122]
Cold	SiO_2_ NPs (10–15 nm)	Wheatgrass (*Agropyron elongatum* L.)	Overcame seed dormancy, enhanced seed germination and seedling weight	Azimi et al. [123]
	Na_2_SeO_4_ NPs (20–35 nm)	Tomato (*S. lycopersicum* L.)	Improved plant growth, chlorophyll, and leaf-relative water contents	Haghighi et al. [124]
	TiO_2_ NPs (~ 20 nm)	Chickpea (*Cicer arietinum* L.)	Enhanced expression of Rubisco- and chlorophyll-binding protein genes	Hasanpour et al. [125]
	ZnO NPs (~ 30 nm)	Rice (*Oryza sativa* L.)	Alleviated chilling stress by regulating the chilling response transcription factors	Song et al. [126
Salinity	SiO_2_ NPs (~ 20 nm)	Tomato (*S. lycopersicum* L.)	Alleviated the effect of salinity on fresh weight, chlorophyll, and photosynthetic rate	Haghighi and Pourkhaloee [127]
	SiO_2_ NPs (~ 12 nm)	Squash (*Cucurbita pepo* L.)	Reduced levels of malondialdehyde (MDA), H_2_O_2_, and electrolyte leakage	Siddiqui et al. [76]
	SiO_2_ NPs (~ 20 nm)	Tomato (*S. lycopersicum* L.)	Suppressed the effect of salinity on germination rate, root length, and fresh weight	Almutairi [128]
	Chitosan NPs (~ 38 nm)	Maize (*Z. mays* L.)	Alleviated the harmful effects of salinity stress	Bruna et al. [129]
	MWCNTs (30–100 nm)	Cabbage (*Brassica oleracea* L.)	Alleviated the harmful effects of salinity stress	Martinez-Ballesta et al. [130]
	ZnO NPs (~ 20 nm)	Sunflower (*Helianthus annuus* L.)	Increased net CO_2_ assimilation rate, sub-stomatal CO_2_ content, and Fv/Fm ratio	Torabian et al. [131]
	Fe_2_O_3_ NPs (~ 50 nm)	Peppermint (*Mentha piperita* L.)	Increased leaf dry weight, phosphorus, potassium, iron, zinc, and calcium contents	Askary et al. [132]
	Fe_2_O_3_ NPs (~ 20 nm)	Wheat (*T. aestivum* L.)	Improved the growth of both root and shoot	Fathi et al. [133]
	ZnO NPs (~ 20 nm)	Wheat (*T. aestivum* L.)	Improved the growth of both root and shoot	Fathi et al. [133]
	SiO_2_ NPs (~ 10 nm)	Cucumber (*Cucumis sativus* L.)	Increased plant germination and growth characteristics	Alsaeedi et al. [134]
	SiO_2_ NPs (20–30 nm)	Soybean (*Glycine max* L.)	Reduced oxidative damage due to expression of antioxidative enzymes	Farhangi-Abriz and Torabian [135]
	Chitosan NPs (~ 25 nm)	Tomato (*S. lycopersicum* L.)	Alleviated the harmful effects of salinity stress	Hernandez-Hernandez et al. [136]
	CeO_2_ NPs (~ 8.5 nm)	Cotton (*Gossypium hirsutum* L.)	Modulated α-amylase activities and ROS homeostasis	Khan et al. [137]
	CeO_2_ NPs (~ 8 nm)	Rapeseed (*Brassica napu*s L.)	Enabled better ability to maintain cytosolic K^+^/Na^+^ ratio	Liu et al. [138]
Drought	TiO_2_ NPs (~ 20 nm)	Wheat (*T. aestivum* L.)	Increased growth, yield, gluten, and starch content	Jaberzadeh et al. [139]
	ZnO NPs (~ 20 nm)	Soybean (*G. max* L.)	Increased germination percentage and rate, decrease in fresh and dry weights	Sedghi et al. [67]
	Fe_2_O_3_ NPs (20–100 nm)	Sunflower (*H. annuus* L.)	Counteracted drought stress with no effect on proline and total amino acids	Martinez-Fernandez et al. [140]
	TiO_2_ NPs (10–25 nm)	Lin seed (*Linum usitatissimum* L.)	Enhanced chlorophyll and carotenoid content, decreased H_2_O_2_ and MDA contents	Aghdam et al. [141]
	MWCNTs (20–30 nm)	Barley (*Hordeum vulgare* L.)	Boosted seed water absorption and increased seedling water content	Karami and Sepehri [142]
	CeO_2_ NPs (6–24 nm)	Soybean (*G. max* L.)	Enhanced growth, development, and yield	Cao et al. [143]
	Fe NPs (40–53 nm)	Strawberry (*Fragaria ananassa* L.)	Enhanced acclimation and resistance of plants to drought	Mozafari et al. [144]
Heavy metal	Fe_3_O_4_ NPs (~ 20 nm)	Rice (*O. sativa* L.)	Reduced As transport from the root to the shoot	Huang et al.[145]
	Si NPs (~ 50 nm)	Wheat (*T. aestivum* L.)	Alleviated Cd toxicity by reducing Cd^2+^ uptake and enhancing antioxidative capacity	Ali et al. [146]
	CuO NPs (9–22 nm)	Rice (*O. sativa* L.)	Reduced total As by 23% and 45% in roots and shoots	Wang et al. [147]
	ZnO NPs (30–40 nm)	Rice (*O. sativa* L.)	Improved plant growth and alleviated the toxic effects of Cd	Zhang et al. [148]
	SiO_2_ NPs (~ 100 nm)	Rice (*O. sativa* L.)	Inhibited As uptake into rice suspension cells via improving pectin synthesis	Cui et al. [149]
	TiO_2_ NPs (36–140 nm)	Rice (*O. sativa* L.)	Reduced As toxicity and reduced As bioaccumulation in rice seedlings by 40–90%	Wu et al.[150]
	Au NPs (~ 40 nm)	Rice (*O. sativa* L.)	Suppressed Cd uptake and alleviated Cd toxicity	Jiang et al. [151]
	ZnO NPs (20–40 nm)	Rice (*O. sativa* L.)	Modulated early growth and enhanced physio-biochemical and metabolic profiles	Li et al. [65]
	ZnO NPs (20–30 nm)	Rice (*O. sativa* L.)	Alleviated the As toxicity and decreased the accumulation of As	Yan et al. [45, 152]

### Extreme temperatures

Plants suffer due to extreme temperatures (heat or cold stress) as their growth, development, and productivity are generally compromised under such conditions [153]. Extreme temperature stresses cause slow growth, low germination rate, decreased photosynthetic rate, denaturation of biomolecules, and disintegration of membrane lipids in crops [154]. NPs can alleviate these bad effects primarily by reducing oxidative stress and the overproduction of ROS [155]. TiO_2_ NPs decreased the energy dissipation of nonregulated PS II and increased the energy dissipation of regulated photosystem II (PS II) during the high-temperature stress in tomato seedlings and finally stimulated the photosynthesis system [119]. The application of TiO_2_ NPs not only prevented membrane damage under cold stress but also alleviated oxidative stress in chickpea (*Cicer arietinum* L.) [156]. NPs promoted crop growth under extreme temperature stress by altering various processes at the physiological, biochemical, and molecular levels (Fig. 4). Moreover, TiO_2_ NPs treatment increased the crop tolerance to cold stress via maintaining the stability of carotenoid and chlorophyll accumulations, inducing the activities of ascorbate peroxidase and catalase [157], and enhancing gene expression of chlorophyll- and Rubisco-binding proteins [125]. Khodakovkaya et al. [158] showed that MWCNTs triggered the response of stresses in crops leading to the upregulation in the expression of different stresses-associated genes including *HSP90*. Similarly, Zhao et al. [118] found that CeO_2_ NPs upregulated the expression of *HSP70* and diminishes the content of H_2_O_2_. The use of ZnO NPs increased the chilling stress-triggered gene expression by modulating the cold response transcription factors in leaves [126].

### Osmotic stress

NPs improve the tolerance of crops to osmotic stresses, e.g., high salinity and drought. The stress caused by over-accumulation of anions of SO_4_^2−^ and Cl^−^ as well as cations of Na^+^, Mg^2+^, and Ca^2+^, commonly known as salinity, limits the production of crops in about one-fifth of the cultivated land around the world [159]. NPs can alleviate the damage caused by high salinity stress to crops in many ways like by restoring the damage to the photosynthesis system and altering the accumulation of metals in crops. Siddiqui and Al-Whaibi [160] found that SiO_2_ NPs treatment increased plant dry weight, seed germination, proline accumulation, and chlorophyll content in squash and tomato plants under NaCl stress. Foliar application of FeSO_4_ NPs not only induced shoot dry weight, chlorophyll content, leaf area, maximum photochemical efficiency of photosystem II (Fv/Fm), and net CO_2_ assimilation rate, but also reduced Na content in leaves of sunflower cultivars under salinity stress [161]. Recent studies on the use of chitosan NPs in tomato [136] and maize [129], MWCNTs in broccoli [130], and Ag NPs in wheat seedlings [121], further reveal the mitigating effect of NPs on high salinity stress. CeO_2_ NPs enhance salt tolerance by enabling better ability to maintain cytosolic K^+^/Na^+^ ratio in cotton [138]. Nanoceria seed priming improves salt tolerance by modulating α-amylase activities and ROS homeostasis in rapeseed [137].

The increasing scarcity of agricultural water has adversely affected agricultural production and destroyed the green crops in the semiarid and arid areas of the world [162]. Huge achievements have been attained to alleviate the bad influences of drought on crop seedlings by using phytonanotechnology in different aspects, such as restoring the plant growth damage caused by severe drought, enhancing water accumulation, and inducing water absorption of seed in crops. Sodium nitroprusside (SN) NPs and MWCNTs enhanced the tolerance to drought stress by increasing seedling water content and boosting seed water absorption in barley [142]. The use of CeO_2_ NPs [143] and micronutrient NPs [163] enhanced crop growth and development exposed to drought stress in soybean.

### Heavy metals

Rapid urbanization and industrialization in recent decades have greatly contributed to soil pollution. Heavy metals, e.g., Arsenic (As), Mercury (Hg), Chromium (Cr), Cadmium (Cd), and Lead (Pb), are among the chief pollutants in soil [164]. Phytonanotechnology, is one of the effective ways to remediate or detoxify dangerous pollutants like toxic heavy metals (HMs) by different routes, such as by decreasing the overproduction of ROS and oxidative stress caused by HMs, reducing their accumulation in food crops, and inhibiting heavy metals-triggered expression of the metal(s) transporter-associated genes in food crops. For example, it was reported that the use of 2.5 mM Si NPs can greatly enhance the tolerance to Cd stress in rice seedlings by minimizing the excessive ROS caused by Cd [165]. Wang et al. [166] also found that Si NPs have a benefit over conventional fertilizers in decreasing the accumulation of HMs in plants. The ZnO NPs reduced the Cd uptake in wheat [167] and decreased the accumulation of As in rice plants [152]. Jiang et al. [151] reported that the application of Au NPs synthesized with melatonin (Mel-Au NPs) alleviated Cd stress in rice, by reducing Cd-generated oxidative stresses and preventing the uptake of Cd. Moreover, Mel-Au NPs treatment inhibited the expression of metal transporter-related genes under Cd stress in rice roots. ZnO NPs-based seed priming modulates early growth and enhances physio-biochemical and metabolic profiles of fragrant rice under Cd toxicity [65].

However, the overuse of NPs may pollute the environment (soil and water) by dispersing from agricultural fields or remediation activities, e.g. fertilizers and pesticides [168]. The high concentration of NPs also harms the growth and development of crops [169]. High levels of NPs have significant effects on gene expression and can induce oxidative stress, resulting in membrane damage, electrolyte leakage, and decreased photosynthetic pigment content in crops [170]. For example, 1 g/kg ZnO or CuO NPs may badly affect the function and structure of photosynthetic machinery in crops and limit the development of roots and shoots [171, 172]. The appropriate dose of NPs is important for the application of phytonanotechnology.

## Nanotechnology in agrochemicals for crop and disease management

Agrochemicals are chemical products that comprise fertilizers, pesticides (insecticides, herbicides, and bactericides), and plant growth regulators used in agricultural practices to improve crop yield and quality [173]. Nanotechnology is widely utilized in agrochemicals, and the details of nanotechnology used in agrochemicals are discussed below.

### Nanotechnology in fertilizers

Fertilizers are necessary for enhancing soil fertility and crop productivity [174]. The environmental restrictions and the incomplete use of nutrients related to the utilization of traditional fertilizers are still big issues for accomplishing sustainability in agricultural systems [175]. Additionally, nanofertilizers can be the best choice to conquer problems like eutrophication and enhance nutrient use efficiency in agriculture [176, 177]. Based on the functions, nanofertilizers can be classified as nano-composite fertilizers, controlled-release fertilizers, or controlled loss fertilizers as combined nano-device to provide different macro- and micro-nutrients with ideal characteristics [178].

The application of nano-composite fertilizers for the controlled release can improve soil health, promote the crop uptake process, regulate rhizosphere microorganisms, and stimulate the productivity and growth of crops [179]. The absorption of NPs not only enhances the content of absorbed elements, but also increases the content of other elements in crops. For example, a study conducted on sandy loam soil-cultivated cucumber seedlings showed that 0.5 g/kg of TiO_2_ NPs exposure produced approximately 34% more P content and 35% more K content than those in the control [180]. Similarly, the influence of ZnO NPs on mineral uptake in cucumber seedlings indicated that ZnO NPs greatly induced uptake of minerals [181]. The uptake of Al in seedlings of lettuce treated by 10 mg/L Fe/Fe_2_O_3_ NPs was increased [182]. A greatly induced absorption of Fe, Zn, S, and Al, and a decreased uptake of P, Mn, and Mg were found in lettuce seedlings when treated with 10 or 20 mg/L Cu/CuO NPs [182]. Similarly, 1 g/kg CeO_2_ exposure to soybean grown in soil induced the accumulations of Cu and P, while it reduced the content of Ca in pods [183]. Nanocalcite (40% CaCO_3_) with nano Fe_2_O_3_ (1%), MgO (1%), and SiO_2_ (4%) remarkably increased the intake of P with micronutrients Mn and Zn, and enhanced the Fe, Ca, and Mg uptake [184]. The Zn, Fe, Ca, and K contents increased after the seedlings were treated with Au NPs in wheat [185]. The supplementation of ZnO NPs with other fertilizers in the Zn deficient soil enhanced the productivity of barley by 91% compared to the control and increased nutrient use efficiency, while the conventional bulk ZnSO_4_ enhanced productivity by only 31% compared to the control [186]. Moreover, NPs with different hydrophobic properties have different changes in the content of elements in crops. Three different types (hydrophobic, hydrophilic, and unmodified) of TiO_2_ NPs exposure to the seedlings have been reported to influence the mineral uptake in basil (*Ocimum basilicum* L.). At the concentration of 0.5 g/kg treatment, the hydrophobic NPs induced Mn content by 339%, the hydrophilic NPs induced the increase of Fe content by 90%, and the unmodified ones increased the Cu by 104%) and Fe by 90% [83]. Furthermore, nano-composite fertilizers showed helpful influences on rhizosphere microorganisms by inducing the secondary metabolite production [187, 188], improving the plant growth [189], and assisting the colonization on the root surface.

The application of controlled-release fertilizers, e.g., porous NMs, greatly improves the uptake process in crops by adjusting the demanded release [190]. A carbon-based NM, graphene oxide film, can extend the release of KNO_3_, which minimizes loss by runoff and leaching, and prolongs the time of effect [191]. Numerous studies have revealed that the decreasing size of NMs is beneficial to the increase of the surface mass ratio of particles. Various nutrient ions can be desorbed and adsorbed steadily and slowly for an extended period of time [192]. For example, the use of ‘controlled release fertilizer’ not only increased the wheat production and soil residual mineral nitrogen by 6% and 10%, but also reduced nitrogen leaching and runoff loss by 25% and 22%, respectively as compared to conventional fertilizers [193, 194]. Nano-fertilizers balance the nutrition during the life cycle and ultimately increase the production of crops.

Numerous studies were conducted on this topic, but the research and information on wider capacity are still inadequate. The study of the toxicity of NPs utilized for nano-composite fertilizer production and the safety of different nano-fertilizers applications should be the priority for research. Moreover, a further evaluation of the different effects of nano-fertilizers in soils with diverse physiochemical features is essential to endorse a specific nanofertilizer for a particular soil type or crop.

### Nanotechnology in pesticides

The application of nanotechnology over conventional crop protection, e.g., over-dose and large-scale pesticide use, has rapidly increased to reach higher and better crop production.

### Insecticides

At least 90% of the applied pesticides are either incapable to achieve the goals for effective control of insects or scattered in the environmental systems [195]. This situation not only leads to the deterioration of the environment but also increases the costs of crop production. It is important to note that the occurrence of active ingredients in the formulation at the lowest effective concentration at the target site is necessary to ensure improved protection of crops from an invasion of insects and subsequent loss of crops. Nano-encapsulation and nano-formulation of insecticides have completely changed crop protection. Nano-encapsulation of pesticides is a technology in which the active ingredients of insecticides are coated with various sizes of NMs [195]. Nano-formulation of insecticides includes a few particles, which can be used as insecticide active ingredients, and other engineered nano-structures have beneficial insecticidal features [196]. Nano-encapsulation and nano-formulation of insecticides assist the controlled release and persistence of active ingredients inside crops or in root zones without influencing the efficiency. Conventional formulations of insecticides not only harm non-target organisms, but also limit the water solubility of insecticides, causing increased resistance to target organisms. Nano-encapsulation and nano-formulation help to overcome the above limits [196]. Nano-encapsulation and nano-formulation of insecticides display many valuable features, such as increased thermal stability, crystallinity, solubility, permeability, stiffness, and also biodegradability essential for sustainable agricultural systems [197]. For instance, nanofibers formulation of pheromone in oriental fruit moth (*Grapholita molesta* L.) has no influences on mortality over time, signifying long-time attract-and-kill influence of insecticide and pheromone and a controlled release of active ingredients [198]. Moreover, some studies have delivered suggestions that the nanoformulations of insecticides help the broadening of plant-mediated universal resistance against insects. For instance, the formulations of SiO_2_ nanosphere can enhance the capability of insecticides to attain the cell sap and infiltrate through crops, thus applying the full function to regulate sucking or chewing type insects [199]. Hence, the NMs in insecticides have a marvelous possibility in pest management.

### Herbicides

Weeds are invasive plants that reproduce or grow aggressively outside their original habitat [200]. The chemical ingredients of synthetic and biological sources, which restrain the growth of plants or kill them, are called herbicides [201]. Modern herbicides are frequently synthetic substances of endogenous hormones in varied plants, which can inhibit the development of objective crops. Although weeds are killed by the use of herbicides, occasionally overused herbicide applications largely influence plant growth, which also delivers harmfulness to human beings [202]. Nanotechnology has the ability for the effective transport of biological or synthetic herbicides by using NMs-based agrochemical formulations or nano-sized preparations [202]. The herbicides are loaded on different types of NPs to improve better removal of weeds and enable higher bioavailability. We can utilize the special characteristics (biodegradability, crystallinity, permeability, solubility, stiffness, and thermal stability) of NMs to develop different kinds of nanoherbicides. Nanoherbicides increase the affinity for the target by providing a larger specific surface area. Herbicides encapsulated in nanoscale help to efficient spraying by reducing the splash losses and spray drift. Nanoherbicides are mixed with the particles of soil and can damage weeds or weed seeds. Herbicides, such as triazine and atrazine could be encapsulated to develop effective release to crops [203]. The majority of accessible herbicides just kill aboveground sections of weeds, but do not prevent the growth of the underground viable sections like tubers or rhizomes that function as an origin for the next generation of weeds [204]. The development of particular molecules of the herbicides encapsulated with NPs aims to target receptors in the weed roots, which penetrates the weed roots and achieves sections that prevent the glycolysis process in roots, hence causing the death of specific weeds [205]. Long-term overuse of herbicides can leave their remains in soils and inhibit the growth of subsequent crops, so detoxification of herbicide remains is essential for sustainable development [206]. The detoxification rate of carboxymethyl cellulose NPs to atrazine herbicides is as high as 88% [207]. Thus, nanotechnology has the potential to improve the application range of herbicides and increase the duration of their effect.

### Bactericides

Bactericides are any chemical substance of a synthetic or biological origin, which can inhibit bacterial growth or kill them [208]. The misuse of bactericide has led to the development of multi-drug-resistant bacteria, which is a significant global threat and is one of the biggest challenges for agricultural activities. Nanotechnology-driven innovations provide hope for overcoming this problem [209]. The effectiveness of NPs depends on their interaction with microorganisms. The development of effective NMs requires in-depth knowledge of the biological aspects of microorganisms and the physicochemical properties of NPs. Metallic oxide NPs, such as MgO [210], Al_2_O_3_ [211], MnO, SiO, and TiO [212], ZnO and CuO [213], have been shown to successfully regulate various crops and soil-borne diseases produced by *Ralstonia solanacearum* [210], *Fusarium oxysporum* [211, 212], *Verticillium Dahliae*, *Fusarium solani*, *Monilinia fructicola*, *Colletotrichum gloeosporioides*, *Botrytis cinerea*, and *Alternaria alternate* [213] in various crops. Furthermore, communities of the microorganism of soil have a direct influence on the quality of soil by various processes, such as symbiotic relationships with the decomposition of organic matter, terrestrial crops, and nutrient cycling [214]. Thus, the protection of soil microbial diversity and biomass is the main task for agricultural systems. The metallic oxide NPs, such as CuO and Fe_3_O_4_ NPs, have a big influence on the size and composition of the microbial communities in the soils [215]. Due to the physicochemical properties of NPs, they provide hope for the development of effective antimicrobial agents for the future.

## Conclusions

In the last decade, nanotechnology obtain enormous achievements in the design and synthesis of NMs and their use in therapy, diagnosis, or other medical purposes. Due to high cost or other factors, applications of nanotechnology in crops cannot be widely utilized in agricultural activities or practices. (i) Despite the great improvement of nanotechnology in plant genetics and crop breeding, the delivery of exogenous enzymes or DNA for genome editing is still a tough task. Based on evidence found in plant cells, soft materials, like polymeric nanostructures, and nanogels can be utilized as promising substances to advance novel approaches for genome editing and controlled release of biomolecules in crops. (ii) The troubles of phytonanotechnology can be conquered by encouraging multidisciplinary manners for the synthesis or design of intelligent NMs. To this end, a joint collaborative initiative that merges the complementary professional capabilities of chemists, biochemists, engineers, geneticists, and botanists may reveal a new horizon in phytonanotechnology. (iii) Regarding crop growth and development, current applications propose that more studies are needed for this direction to ameliorate the sustainability of agricultural systems. Future studies involving open-field trials may further benefit to recognizing the mechanism of NPs action on crops.

When used in the agricultural system, these NPs need to be carefully designed, considering their treatment methods (soil or foliar), so that they can have a high impact and ensure a better quality of crops. Meanwhile, excessive use of these NMs may pollute the environment, thus special care must be adopted while working with NMs in plant systems. However, it is an undeniable fact that the positive functions of NPs have shown great efforts to numerous aspects in agricultural systems starting from germination to postharvest.

## Abbreviations

NMs: Nanomaterials
NPs: Nanoparticles
MSNs: Mesoporous silica NPs
CNTs: Carbon nanotubes
QDs: Quantum dots
MNPs: Magnetic NPs
GFP: Green fluorescent protein
MWNTs: Multi-walled nanotubes
SWNTs: Single-walled nanotubes
VNPs: Virus-like NPs
CRISPR: Clustered regularly interspaced short palindromic repeats
Cas: CRISPR-associated proteins
sgRNA: Single guide RNA
RNP: Ribonucleoproteins
ROS: Reactive oxygen species
PS II: Photosystem II
LHCII: Light-harvesting complex II
UV: Ultraviolet
SN: Sodium nitroprusside
